# Association between Periodontitis, Genetic Polymorphisms and Presence of Coronary Artery Disease in Southern Brazil

**DOI:** 10.36660/abc.20180296

**Published:** 2020-02

**Authors:** Luiz Otavio Rocha, Eduarda Rocha, Guilherme de Menezes Succi, Rui Barbosa de Brito Junior

**Affiliations:** 1 Universidade Federal de Santa Maria, Santa Maria, RS - Brazil; 2 Faculdade São Leopoldo Mandic, Campinas, SP - Brazil

**Keywords:** Periodontitis, Polymorphism, Genetic, Coronary Artery Disease, C-reactive Protein, Epidemiology

## Abstract

**Background:**

Periodontitis and coronary artery disease (CAD) share an inflammatory etiology; there is a recent concern regarding the investigation of an association between these two conditions. Current theories indicate that cytokines and proteins have an important role in this process. C-reactive protein and interleukin-6 are inflammatory derivatives produced in the presence of periodontitis and in the pathophysiology of coronary disease. The polymorphisms of CRP + 1444 C > T and IL6-174 G > C are recognized in the literature as being related to CAD.

**Objective:**

This study investigates the association between periodontitis and coronary artery disease, through the presence of PCR and IL-6 polymorphisms.

**Methods:**

We selected 80 patients who underwent diagnostic catheterization in the HU of UFSM. The presence of periodontitis was determined by the Community Periodontal Index, whereas the CAD was established by the medical report. DNA was collected from a saliva sample and the presence of polymorphism was determined by PCR and restriction enzymes. A significance level of 5% was adopted.

**Results:**

The mean age of all participants (p = 0.035, OR 2.65; 95%CI: (1.02-6.87) male gender (p = 0.012, OR 3.37; 95% CI: (1.28- (p = 0.013, OR 3.66; 95% CI: (1.27-10.5)), PCR polymorphism + 1444C > T (p = 0.001, OR 6.37; 95% CI:, (2.25-17.9)) and IL6 -174 G > C polymorphism (p = 0.025, OR 2.87, 95% CI: (1.09-7.55)) were statistically associated with the presence of CAD. Age > 60 years and presence of the PCR +1444 C > T polymorphism remained independently associated with CAD after adjustment by logistic regression.

**Conclusions:**

The presence of the PCR + 1444 C > T polymorphism in this study was independently associated with the presence of coronary artery disease.

## Introduction

Periodontitis is a chronic inflammatory disease, induced by biofilm consisting of gram-negative bacteria, leading to the destruction of the tissues supporting the tooth,^1-3^ with high prevalence worldwide.^4^ The presence of periodontitis triggers the immune system, locally and at distant sites, high concentrations of cytokines and proinflammatory proteins, as well as bacteremia and endotoxemia caused by the bacteria that populate the disease site.^5^

Coronary artery disease (CAD) is a chronic, complex, multifactorial, continuous inflammatory condition that consists in the accumulation of atheromatous plaques in the intima layer of the coronary arteries,^6-9^ being responsible for acute coronary syndromes, the main cause of death in the Western hemisphere.^6,7^

Inflammation plays a very important role in both periodontitis and CAD. In order to associate these two conditions, two biologically plausible theories were developed, focusing on the direct and indirect action of the oral bacteria present in periodontitis with the inflammatory and pro-atherogenic mediators.^1,2,4,10-14^

Based on the inflammatory and immunological theories, several studies have been conducted aiming to establish this association through the genetic factor, the presence of polymorphisms in the genes that express the production of these factors and are associated to periodontitis, such as C-Reactive Protein (CRP) and interleukin-6 (IL-6).^15-19^

The simple nucleotide polymorphism (SNP) of the IL-6 promoter gene may affect the production and expression of this cytokine; consequently, this change in serum levels may result in a relevant biological response.^18^ The association between the SNP variant -174 G > C (rs1800795) and the increased risk of inflammatory diseases such as CAD has been previously demonstrated.^17,20^

Studies point to the SNP rs1136804, also represented as 3 ‘UTR +1444 C > T, as the polymorphism with greater associations with CAD.^15,17^

The high prevalence of periodontitis, as well as the high risk of mortality from CAD and a scarcity of studies in this area led to the study of the association between periodontitis and coronary artery disease, by assessing the presence of PCR and IL-6 polymorphisms.

## Methods

The sample consisted of 80 patients (mean age 60.5 ± 10.5) who underwent diagnostic cardiac catheterization in the hemodynamic laboratory of the University Hospital of *Universidade Federal de Santa Maria* (HUSM) from September 1, 2010 to March 30, 2011 and who agreed to participate in the study by signing the informed consent form.

Were excluded for the sample Patients who were smokers, diabetics, those with autoimmune diseases and those with fewer than two teeth present in one of the sextants, as well as those who did not agree to participate in the study.

The study was approved by the Ethics and Research Committee of the São Leopoldo Mandic Dental Research Center on 11/26/2014, CAAE: 35879614.7.0000.5374.

Periodontal examination was performed by the researcher, using the Community Periodontal Index (CPI) and Periodontal Insertion Loss Index (PIP), as recommended by the World Health Organization.^21^ The use of this index was justified by the partial immobilization of the patient in bed and the short time in which it was available to the examiner. Patients with a CPI score of 3 or 4 and a PIP score above 1 were considered as having periodontitis.

DNA was extracted from a saliva sample obtained through a mouthwash with 3% glucose solution for 1 minute, and then this material was deposited in capped, sterile test tubes and frozen at -20°C.^22^

The diagnosis of CAD was performed through cardiac catheterization, via femoral or radial access, performed by the physicians of the hemodynamic laboratory of the Santa Maria University Hospital. Patients with a positive medical report for the presence of coronary stenosis were considered as having CAD.

### Genotyping

DNA was extracted from saliva samples had using a genomic DNA extraction kit (Norgen Biotek Corp, Canada), and gene amplification was performed by the polymerase chain reaction using the following primer pairs for the +1444 PCR: forward 5 ‘- AGCTCGTTAACTATGCTGGGGCA-3’ and reverse 5 - CTTCTCAGCTCTTGCCTTATGAGT-3 ‘, with an annealing temperature of 60 °C and for IL-6 -174: forward 5’-AACCTAATTCTACCCCCCTTGG-3 and reverse 5’-TCAGAGGCAGCCGAAGAGTT-3’, with an annealing temperature of 59°C.17

The amplification was carried out using one cycle at 95°C for 3 min, 29 cycles at 95°C for 1 minute, with annealing temperature varying as cited, for 1 minute, 72°C for 1 minute and a of 72°C cycle for 10 minutes, performed in an automatic cycler.

Polymerase chain reaction results were then digested by Sdul (Thermo Fischer Scientific, Massachusetts, USA) for +1444 PCR and HaeIII (Thermo Fischer Scientific, Massachusetts, USA) for IL-6 -174, using the specified amounts. The resulting fragments were identified by silver-stained 8% polyacrylamide gel electrophoresis. Genetic analyses were performed in July 2017 at the São Leopoldo Mandic molecular biology laboratory, Campinas, SP.

### Statistical analysis

The variables were categorized as follows: CAD as present or absent, periodontitis as present or absent, age ≥ 60 years or ≤ 59 years, gender as male or female, ethnicity as white or non-white, overweight and obesity when BMI was ≥ 25 and ≤ 24 and polymorphisms by the presence or absence of the risk allele. The outcome was the presence or absence of CAD.

A significance level of 5% (p < 0.05) was used. The sample was divided into two groups, according to the presence of CAD; case group with 52 patients (mean age of 62.8 ± 11.2) and control group with 28 patients (mean age of 56.2 ± 8.8).

The distribution of the genotypes of the two SNPs was tested for the Hardy-Weinberg equilibrium by chi-square test (x2), the collected variables were crossed with the chi-square with odds ratio (OR) and 95% confidence interval (95%CI)

The variables that showed statistical significance were submitted to adjustment through a binary logistic regression with CAD as outcome and 95% CI.

All data collected were treated using the SPSS^(©)^ version 25 software (IBM^(©)^ Corp., New York, NY).

## Results

Table 1 shows the association between the studied variables and the presence of coronary artery disease, and it was observed that the age categorized as the cut-off of the mean of all participants, 60 years (p = 0.035), male gender (p = 0.012) and patients with periodontitis (p = 0.013) were statistically related to the presence of CAD. Patients with the + 1444 C > T polymorphism, with the presence of risk allele T (p = 0.001), as well as those with the IL6 -174 G > C polymorphism, with risk allele C (p = 0.025), were associated with the presence of CAD.

**Table 1:** Variables analyzed by chi-square with CAD as outcome

Analyzed variables		CAD n = 80		OR	CI (95%)		p
		Present (%)	Absent (%)		Min.	Max.	
Age	≥ 60 years	31 (59.6)	10 (35.7)	2.65	1.02	6.87	0.04
	≤ 59 years	21 (40.4)	18 (64.3)				
Gender	Male	32 (61.5)	9 (32.1)	3.37	1.28	8.9	0.01
	Female	20 (38.5)	19 (67.9)				
Overweight and obesity	BMI ≥ 25	30(57.7)	22 (78.6)	2.68	0.93	7.73	0.35
	BMI ≤ 24	22 (42.3)	6 (21.4)				
Ethnicity	White	45 (86.5)	22 (78.6)	1.75	0.52	5.84	0.06
	Non-White	7 (13.5)	6 (21.4)				
Periodontitis	Present	26 (50%)	6 (21.4)	3.66	1.27	10.5	0.01
	Absent	26 (50%)	22 (78.6)				
CRP +1444 C>TRS 1130864	T Allele	43 (82.7)	12 (42.9)	6.37	2.25	17.9	0.001
	Non T allele	9 (17.3)	16 (57.1)				
IL6-174 G>CRS 1800795	C Allele	30 (57.7)	9 (32.1)	2.87	1.09	7.55	0.029
	Non C allele	22 (42.3)	19 (67.9)				

CAD: coronary artery disease; OR: Odds Ratio, CI: confidence interval, significant when the range does not contain the unit. significant p-value < 0.05.

Source: The author.

A binary logistic regression analysis was carried out, in which all the significant independent variables were included in the bivariate analyses in a direct way, with the objective of verifying which of them would be predictors of the dependent variable, i.e., the presence of CAD.

The result of this adjustment (Table 2) showed that the presence of CAD was still associated with age > 60 years (p = 0.029) and the presence of the PCR polymorphism +1444 C > T (p = 0.014).

**Table 2:** Variables adjusted by logistic regression

Variables	Chi-square				Logistic regression			
	p	OR	CI (95%)		p	OR	CI (95%)	
			Min.	Max.			Min.	Max.
Age ≥ 60 years	0.04	2.65	1.02	6.87	0.02	3.6	1.14	11.3
Male gender	0.01	3.37	1.28	8.91	0.37	1.71	0.51	5.67
Periodontitis present	0.01	3.66	1.27	10.5	0.16	2.47	0.68	8.9
CRP +1444 polymorphism	0.001	6.37	2.25	17.9	0.014	4.31	1.34	13.8
IL6 -174 polymorphism	0.029	2.87	1.09	7.55	0.06	2.94	0.94	9.19

Significant p-value < 0.05; OR: Odds Ratio; CI: confidence interval, significant when the range does not contain the unit.

Source: The author.

## Discussion

Periodontitis is a chronic, multifactorial inflammatory disease, resulting from a series of dysbiosis processes, activating the production of proteins and proinflammatory cytokines and signaling processes, thus, according to recent epidemiological, interventional and functional studies, establishing a causal association with the development of coronary artery disease.^10-12,16^

The dichotomized age over 60 years and the male gender were also statistically associated with a higher probability of presenting CAD. It is noteworthy that age and male gender are already known risk factors for coronary artery disease and periodontitis,^23^ so much so that many studies adjust for these factors only when analyzing atherosclerosis. However, regarding the association between periodontitis and CAD, with the influence of other independent covariates, it was decided to keep them, in view of the contribution of adjustments in logistic regression models.

The presence of periodontitis in the bivariate analysis was significantly associated with CAD (p = 0.013; OR = 3.66 CI (95%) 1.27-10.5). This association has been studied for decades. A cross-sectional study with 60,174 participants that analyzed the association between periodontitis and CAD found a statistically significant association between the two conditions with an odds ratio of 1.59 and CI (95%) between 1.31 and 1.81, after adjustment for confusion factors.^23^

In the present study we verified the association between periodontal inflammation and polymorphisms (IL6 and CRP) aiming to verify its possible association with CAD. We observed a strong association (p = 0.001) between the presence of the PCR + 1444 C > T polymorphism, risk allele T and the case group with OR = 6.37; (95%) 2.25 - 17.9, which contradicts authors who analyzed five studies, totaling 18,637 participants, where the PCR + 1444 C > T polymorphism was adjusted for confounding factors and compared regarding the presence of CAD, but found no association between this polymorphism and coronary disease.^24^ It is possible to infer that this association may arise from CRP serum levels maintained by chronic periodontitis over several years.

The presence of this association is corroborated by studies comparing the size of atherosclerotic plaques in relation to the PCR polymorphism at this SNP (+1444 C > T) in 196 patients with CAD from a database of studies evaluating the use of nitrates (ENCORE) in Switzerland, concluding that the carriers of this polymorphism were independently prone to larger plaque volumes.^15^

These results lead us to believe that somehow the presence of this polymorphism, probably through CRP serum levels, acts directly in the atherosclerotic process, as indicated by recent studies.^25,26^

The IL6 -174 G > C polymorphism was statistically associated with the presence of CAD (p = 0.025, OR = 2.87 CI (95%) 1.09-7.55). These findings are in agreement with authors who investigated the association of this polymorphism with the risk of CAD in 484 Chinese individuals, and found that the IL6 -174 G > C polymorphism was positively associated with the risk of CAD (p = 0.001 OR = 2.18 CI 95%) 1.26 - 3.77), in agreement to our findings, but with a higher statistical significance.^18^

In a recent study, 280 patients were analyzed in an attempt to correlate five polymorphisms, among them IL6 -174 G > C, with CAD in a population from northern India. In this analysis, the authors did not find any statistical significance regarding this polymorphism and CAD.^20^ Similarly, Brazilian authors analyzed 200 patients with acute coronary syndrome and their association with the presence of the IL6 -174 G > C polymorphism in Pernambuco, Brazil, and found no significant association between the presence of the risk alleles and acute coronary syndrome.^27^

The variables that were significant were included in a bivariate logistic regression model with the purpose of adjusting the independence of these associations, verifying which of them would predict CAD. The most uniform model was the one that remained independently associated with CAD, age > 60 years and the presence of the +1444 C > T PCR polymorphism (table 2).

Considering the limitation of the type of study (case-control) where the information about the exposure or factor is obtained after the occurrence of the disease and there is no way to differentiate the chronology between the exposure and the disease onset, to determine a direct causal association between periodontitis and CAD becomes more difficult. The fact that this association is present may indicate preventive and curative treatments for the control of periodontitis, aiming to reduce the group of factors that contribute to the formation and development of CAD, besides the known risk factors.

## Conclusions

Based on the analyzed data, it can be concluded that the age over 60 years and the presence of the PCR + 1444 C > T polymorphism were independent predictors associated with coronary artery disease.

The presence of periodontitis and male gender did not remain associated with CAD after adjusting by logistic regression.
